# Physiologically Based Pharmacokinetic Modeling in Pregnant Women Suggests Minor Decrease in Maternal Exposure to Olanzapine

**DOI:** 10.3389/fphar.2021.793346

**Published:** 2022-01-19

**Authors:** Liang Zheng, Hongyi Yang, André Dallmann, Xuehua Jiang, Ling Wang, Wei Hu

**Affiliations:** ^1^ Department of Clinical Pharmacology, The Second Hospital of Anhui Medical University, Hefei, China; ^2^ Department of Clinical Pharmacy and Pharmacy Administration, West China School of Pharmacy, Sichuan University, Chengdu, China; ^3^ Pharmacometrics/Modeling and Simulation, Research and Development, Pharmaceuticals Bayer AG, Leverkusen, Germany

**Keywords:** olanzapine, PBPK, pregnancy, metabolic enzymes, pharmacokinetics

## Abstract

Pregnancy is accompanied by significant physiological changes that might affect the *in vivo* drug disposition. Olanzapine is prescribed to pregnant women with schizophrenia, while its pharmacokinetics during pregnancy remains unclear. This study aimed to develop a physiologically based pharmacokinetic (PBPK) model of olanzapine in the pregnant population. With the contributions of each clearance pathway determined beforehand, a full PBPK model was developed and validated in the non-pregnant population. This model was then extrapolated to predict steady-state pharmacokinetics in the three trimesters of pregnancy by introducing gestation-related alterations. The model adequately simulated the reported time-concentration curves. The geometric mean fold error of C_max_ and AUC was 1.14 and 1.09, respectively. The model predicted that under 10 mg daily dose, the systematic exposure of olanzapine had minor changes (less than 28%) throughout pregnancy. We proposed that the reduction in cytochrome P4501A2 activity is counteracted by the induction of other enzymes, especially glucuronyltransferase1A4. In conclusion, the PBPK model simulations suggest that, at least at the tested stages of pregnancy, dose adjustment of olanzapine can hardly be recommended for pregnant women if effective treatment was achieved before the onset of pregnancy and if fetal toxicity can be ruled out.

## Introduction

The peak incidence of many psychiatric illnesses such as schizophrenia in women occurs during their reproductive years (Kulkarni et al., 2015). The prescription of second-generation antipsychotics (SGAs) to pregnant women has been steadily increasing in the last 20 years. The latest statistics from ten countries show that up to 2% of pregnant women use SGAs (Reutfors et al., 2020). Though concerns about the safety of antipsychotics during pregnancy persist, some large-scale clinical studies in recent years suggested that exposure to SGAs does not confer an increased risk of congenital malformations (Huybrechts et al., 2016; Damkier and Videbech, 2018). Given the severe consequences without pharmacotherapy, off-label use of antipsychotics during pregnancy may be inevitable. Except for concerns about fetal safety, clinicians often face another major challenge, i.e., optimizing dosage regimens to obtain effective maternal treatment.

The potential benefits of therapeutic drug monitoring (TDM) to optimize pharmacotherapy are particularly obvious in psychiatry and neurology. The TDM task force of the German Society of Neuropsychopharmacology and Pharmacopsychiatry (Arbeitsgemeinschaft für Neuropsychopharmakologie und Pharmakopsychiatrie [AGNP]) had given many antipsychotics a high recommendation strength for conducting TDM (Hiemke et al., 2018). For olanzapine, a reference concentration range of 20–80 ng/ml was recommended. On the other hand, pregnancy introduces conspicuous changes in various anatomical, physiological, and biological properties, for instance, organ blood flow and hormone levels. Those alterations will influence drug disposition and further their system exposure (Kazma et al., 2020). According to a comprehensive review, gestation-associated changes in pharmacokinetics widely exist (Pariente et al., 2016). Blood concentrations of commonly prescribed antipsychotics perphenazine, quetiapine, and aripiprazole decrease sharply in late pregnancy, suggesting effective treatment may not be achieved in this period with the pregestational dosing regimen (Westin et al., 2018). However, a paucity in complete pharmacokinetic reports makes it challenging to implement dose adjustment for pregnant women.

Physiologically based pharmacokinetic (PBPK) modeling serves as a critical pharmacometrics tool to make reliable pharmacokinetic predictions in special populations. The number of new drug application submissions to the US Food and Drug Administration (FDA) that included PBPK modeling for pediatric drug development has continued to grow over the past decade (Corriol-Rohou and Cheung, 2019) and the role of PBPK modeling for pregnant women in a regulatory context has been discussed recently (Coppola et al., 2021; Green et al., 2021). Since PBPK is a mechanism-based modeling method, the combined effects of multiple gestation-related physiological changes on drug disposition can be incorporated. The confidence in current pregnant modeling tools is restricted by a lack of robust data around the understanding of some metabolic enzymes and transporters and how gestation and genotypes affect drug exposure jointly (Abduljalil and Badhan, 2020). Despite these shortages, PBPK modeling seems promising to address an imperative query: whether dose adjustment is required during pregnancy. In a previous study, we proposed optimized dosage regimens of quetiapine and aripiprazole for the pregnant population using PBPK modeling and simulation (Zheng et al., 2021).

Olanzapine undergoes extensive metabolism in the liver. Several enzymes, namely cytochrome P450 (CYP) 1A2, 2C8, 3A4, flavin monooxygenase 3 (FMO3), and glucuronyl-transferase (UGT) 1A4, are responsible for the metabolism. Six metabolic pathways of olanzapine have been identified, and some metabolic pathways such as N-demethylation are mediated by different enzymes (Supplementary Figure S1) (Kassahun et al., 1997; Korprasertthaworn et al., 2015). UGT1A4 catalyzes 10-N-glucuronidation and 4′-N-glucuronidation, whose metabolites account for 23% of an administered oral dose in non-pregnant adults (Kassahun et al., 1997). The precise proportions of other metabolites generated by oxidases have not been determined. The *in vivo* pharmacological effects are believed to be derived mainly from the parent drug (Hiemke et al., 2018). Though the principal metabolic enzyme CYP1A2 reveals a sharp reduction in its metabolic ability during pregnancy, plasma concentrations of olanzapine appear to be not markedly changed according to therapeutic drug monitoring data reported from Norwegian hospitals (Tracy et al., 2005; Westin et al., 2018). Thus, this study aims to develop a whole-body PBPK model for olanzapine to evaluate the change in systemic exposure of olanzapine throughout pregnancy. The results of this study will be beneficial for rational antipsychotic medication in this vulnerable population.

## Materials and Methods

### Software and General Workflow

We used Open Systems Pharmacology Suite incorporating PK-Sim^®^ and MoBi^®^ (https://github.com/Open-Systems-Pharmacology) to implement the modeling work. The software is freely distributed under the GPLv2 license (Lippert et al., 2019). Parameter identification and sensitivity analysis were conducted within PK-Sim^®^. The reported plasma time-concentration data were digitized using WebPlotDigitizer version 4.2 (Ankit Rohatgi, Austin, United States). Plot creation and statistical analysis were conducted with OriginPro^®^ (OriginLab, Northampton, United States).

**FIGURE 1:**
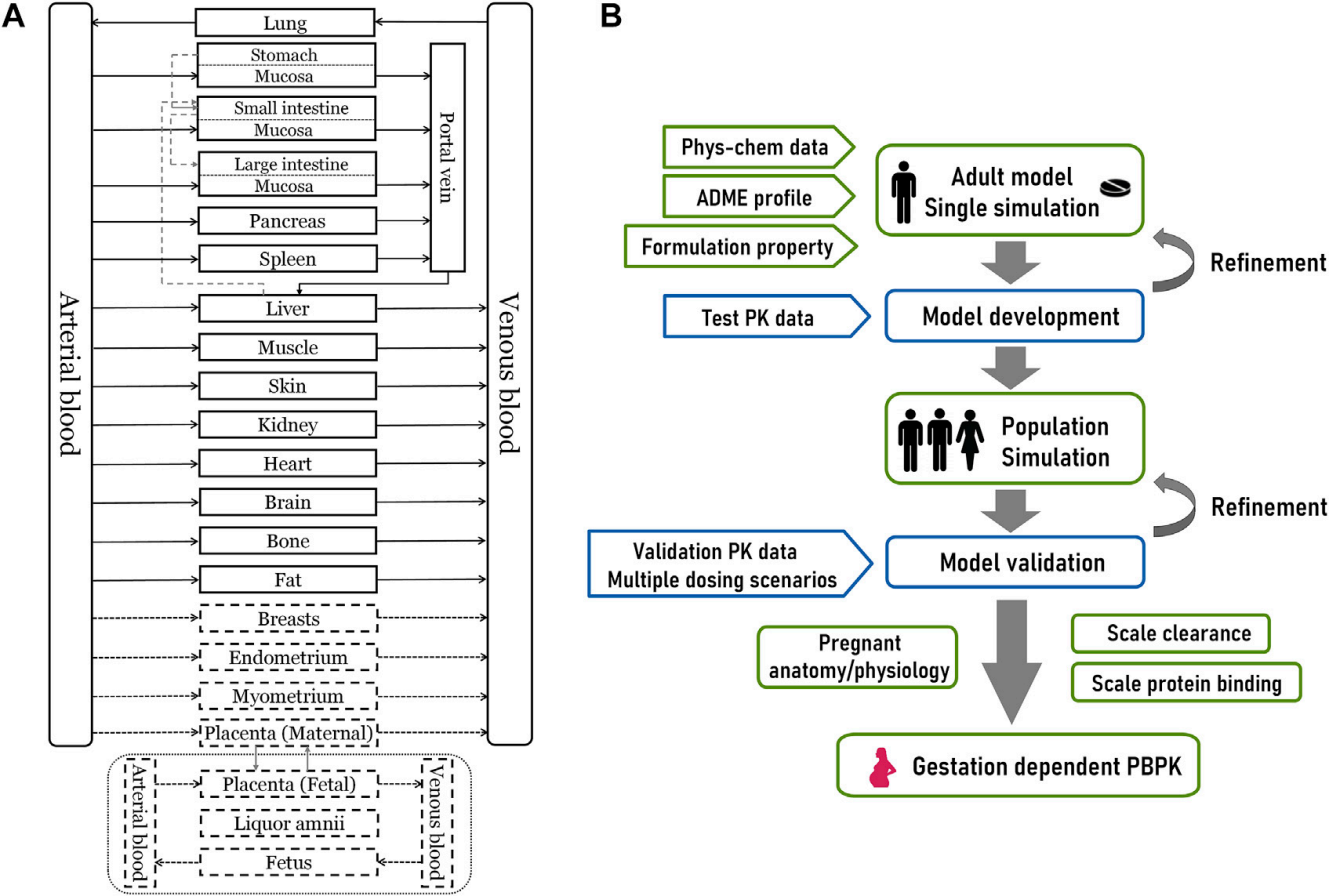
The overall design of this modeling study **(A)** A 27-compartment physiological model of pregnant women in Mobi^®^. The dotted portion is nine gestation-specific compartments **(B)** The schematic diagram for PBPK modeling workflow. Phys-chem, physicochemical; ADME, absorption, distribution, metabolism, and excretion.

An overview of the 27-compartment pregnancy model structure and general modeling workflow is depicted in Figure 1. As the first step, we constructed the adult PBPK model of olanzapine using the default 18-compartment model structure designed for small molecules (Willmann et al., 2003). The model was validated with pharmacokinetic data under multiple scenarios, including studies in pediatrics and smokers. The validated model was then scaled to the pregnant population after modifying gestation-related anatomy/physiology and changes in protein binding, metabolism, and renal excretion.

### Clinical Data

We searched and extracted the published clinical pharmacokinetic data of olanzapine and classified them into the test set and validation set. To avert differences caused by pharmaceutical preparations, we excluded studies not using the reference-listed drug (Zyprexa^®^) or generic drugs proved to be bioequivalent. The detailed subject demographics and dosing information are provided in Supplementary Table S1. The test set used to assist modeling is a clinical drug-drug interaction (DDI) study conducted in adult males. This study reported single-dose (10 mg) oral pharmacokinetic profiles with or without co-administration with the strong CYP1A2 inhibitor fluvoxamine (Wang et al., 2004).

### Model Development and Evaluation

The input compound-specific parameters for model development are listed in Table 1. Lipophilicity (measured as logP value) and intestinal transcellular permeability were optimized using the Monte-Carlo algorithm. Tissue-to-plasma partition coefficients and cellular permeabilities were calculated by the Schmitt and Charge-dependent Schmitt method, respectively. For *in vivo* clearance, we first determined the contributions of each pathway to the total clearance. Olanzapine was eliminated primarily by hepatic metabolism, while direct renal excretion (f_R_), composed of glomerular filtration and tubular secretion, accounted for only 7% of an administered oral dose (Kassahun et al., 1997). The dose fraction metabolized by UGT1A4 was set to 0.23, corresponding to the proportion of recovery as glucuronide conjugates from urine and feces (Kassahun et al., 1997). The contribution of CYP1A2 was reckoned to be 0.50 based on data from the abovementioned DDI study according to the Rowland-Matin equation (Eq. 1) (Elsby et al., 2012). A validated PBPK model of fluvoxamine developed by Britz et al. was used for model development (Britz et al., 2019).

$$\frac{AUC_{i}}{AUC}=\frac{F_{g,\;\;i}}{F_{g}}\times \frac{1}{\frac{\sum f_{m}\times f_{m,\;\;CYP1A2}}{1+\frac{I_{u}}{K_{i}}}+\left(1-\sum f_{m}\times f_{m,\;\;CYP1A2}\right)}\;$$ (1)

**TABLE 1:** Summary of input compound parameters of olanzapine PBPK model.

Parameters	Values/Methods	Source
LogP	2.85	modified from reported values (2.77, 2.89) Ela et al. (2004), Urmila Sri Syamala (2013)
f_u_ (non-pregnant adults)	0.07	drug label^b^
MW (g/mol)	312.4	—
dissociation type	Monoprotic base	—
PKa	7.24	Callaghan et al. (1999)
solubility (μg/ml)	145.4	Urmila Sri Syamala (2013)
dissolution time (50% dissolved, min)	10	Ding (2012)
transcellular permeability (cm/min)	3.85E-6	parameter identification
partition coefficients	Schmitt	Schmitt (2008)
cellular permeabilities	Charge dependent Schmitt	Schmitt (2008)
CL_int,CYP1A2_ (L/h)	26.67	fitted to f_m,CYP1A2_
CL_int,CYP3A4_ (L/h)	0.82	fitted to f_m,CYP3A4_
CL_int,CYP2C8_ (L/h)	2.14	fitted to f_m,CYP2C8_
CL_int,FMO3_ (L/h)	4.05	fitted to f_m,FMO3_
CL_int,UGT1A4_ (L/h)	20.06	fitted to f_m,UGT1A4_
f_m,CYP1A2_ ^a^	0.50	calculated
f_m,UGT1A4_ ^a^	0.23	Kassahun et al. (1997)
f_m,CYP3A4_/f_m,CYP2C8_/f_m,FMO3_ ^a^	0.067	assumed
GFR fraction	1.0	assumed
CL_TSspec_ (L/min)	0.31	fitted to f_R_
f_R_	0.07	Kassahun et al. (1997)

logP, lipophilicity; MW, molecular weight; pKa, acid dissociation constant; CL_int_, intrinsic clearance; f_m_, fraction metabolized by a specific enzyme; f_u_, fraction unbound; GFR, glomerular filtration fraction; CL_TSspec_, specific clearance by tubular secretion; f_R_, fraction excreted via kidney.

a please note that these parameters are not model input parameters, but model output values calculated from the simulated pharmacokinetics and that they differ in pregnant women.

b official drug label of Zyprexa^®^ (https://www.accessdata.fda.gov/drugsatfda_docs/label/2009/022173lbl.pdf).

Further details of using Eq. 1 to calculate the dose fraction metabolized by CYP1A2, including a description of the variables in this equation, is provided in the supplementary material. Contributions of secondary enzymes involved in olanzapine metabolism including CYP2C8, CYP3A4, and FMO3 were roughly estimated to be equal. Olanzapine exhibited dose-dependent pharmacokinetics, and metabolic saturation was not observed; therefore, we used first-order processes to define metabolism according to .

$$v=CL_{\mathrm{int},E}\times S$$ (2)

*CL*_*int,E*_ is the normalized intrinsic clearance (L/min) obtained by fitting to the test set data and to the fraction metabolized through each enzyme (f_m_) determined before. S is substrate amount (µmol) and v the reaction rate (µM/min).

Sensitivity of the final model to single parameters (local sensitivity analysis) was measured as relative change of area under the concentration-time curve (AUC, ng∙h/mL) after a single oral dose or AUC from time of the last dose administration to infinity after multiple administrations. Parameters were included in the analysis if they were optimized or associated with optimized parameters or if they might have a substantial impact due to calculation methods. Sensitivity to a parameter was calculated according to .

$$\mathrm{Sensitivity}=\frac{\mathrm{\Delta AUC}}{\mathrm{AUC}}\times \frac{p}{\mathrm{\Delta p}}\;$$ (3)

where ΔAUC = change of the simulated AUC, AUC = simulated AUC with the original parameter value, Δp = change of the examined model parameter value, and *p* = original model parameter value. A sensitivity value of +1.0 denotes that a 10% increase of the examined parameter causes a 10% increase of the simulated AUC (Hanke et al., 2018).

For model evaluation, population simulations to the validation set were performed. The simulated time-concentration curves were compared with the observed ones. The geometric mean fold error (GMFE) for observed C_max_ (ng/ml), t_max_ (h), and AUC as an index of quantitative assessment was calculated according to .

$$GMFE=10^{\left(\sum \left|\mathrm{lg}\left(\frac{predicted\;PK\;parameter}{observed\;PK\;parameter}\right)\right|\right)/n}$$ (4)

where n is the number of simulated studies. The predicted and observed PK parameters used geometric or arithmetic means depending on reports of clinical studies. If not available, the parameters were calculated by non-compartment analysis using the concentration data. A GMFE value less than two suggests satisfactory predictive performance (Britz et al., 2019). A more detailed description of model development and evaluation is provided in the Supplemental Material.

### Extrapolation to the Pregnant Population

The pregnancy model with anatomic physiological alterations was developed by Dallmann et al. and described in detail in several publications (Dallmann et al., 2017a; Dallmann et al., 2017b). The model was built in MoBi^®^ and exported to PK-Sim^®^ for population simulation. The following compound-related parameters were considered for adjustments in the pregnancy model. The unbound fraction in plasma during pregnancy was deduced from the base value measured in non-pregnant adults according to Eqs 5, 6 (Dallmann et al., 2017b).

$$f_{u}=\frac{1}{1+K_{A}\times P/MW_{\mathrm{albumin}}}$$ (5)

$$P\left(\frac{g}{L}\right)=14.7\exp\left(-0.0454\mathrm{FW}\right)+31.7$$ (6)

where f_u_ is the plasma unbound fraction of olanzapine, *K*_A_ is the equilibrium association constant (µmol^−1^), P represents the albumin concentration in plasma in a specific fertilization week of pregnancy (µmol/L), MW_albumin_ is the molecular weight of albumin (g/mol), and FW denotes fertilization weeks, which is calculated by subtracting 2 weeks from the gestational week. To calculate the fraction unbound in pregnancy, *K*_A_ was first calculated for non-pregnant adults by re-arranging Eq. 1 using a value of 0.07 for the fraction unbound in non-pregnant adults (see Table 1) and 31.7 g/L for the albumin concentration (Dallmann et al., 2017a). Thereafter, the fraction unbound in pregnancy was calculated from Eqs 5, 6 using the same *K*_A_ value for pregnant women as for non-pregnant adults.

Renal clearances (glomerular filtration, tubular secretion, and renal plasma clearance) are technically interpreted as passive transport processes in the model. Their values as listed in Table 1 are normalized to the volume of kidney and can be left unchanged in pregnancy (Dallmann et al., 2017b). Drug metabolism was modified by activity change of metabolic enzymes taking fertilization week as the independent variable.

#### CYP1A2

CYP1A2 activity changes during pregnancy can be reflected by changes in the apparent clearance of caffeine which is described by Eq. 7 (Dallmann et al., 2018a).

CYP1A2 activity change (%)=0.0291FW2−2.77FW  (7)

#### UGT1A4

**FIGURE 2:**
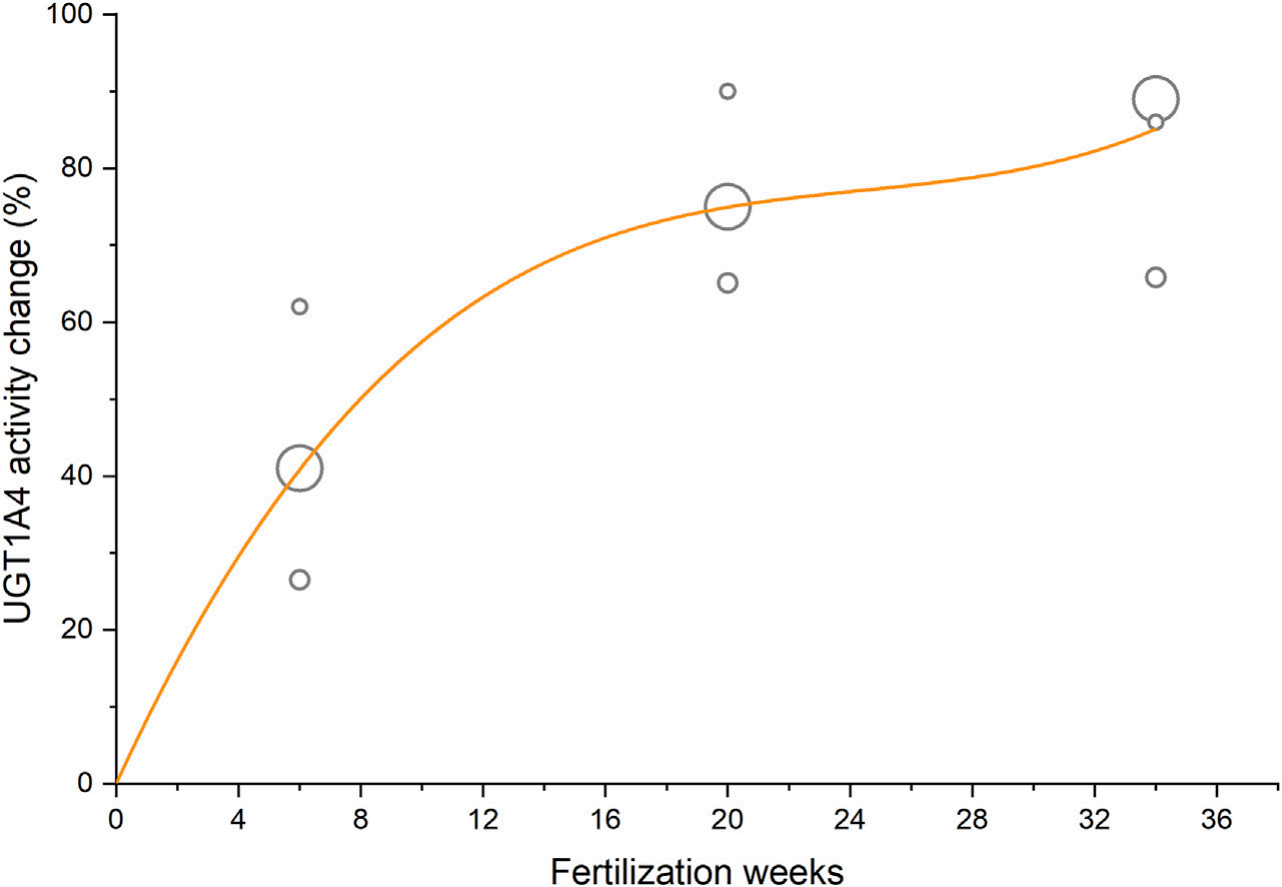
Activity change of UGT1A4 during pregnancy taking lamotrigine apparent clearance as an indicator. The circle area reflects the sample size of clinical studies, and the curve represents the fitted regression equation.

The antiepileptic drug lamotrigine was mainly eliminated through N-glucuronidation which is predominantly catalyzed by UGT1A4 *in vivo* (Wang et al., 2015). Previous studies reported the apparent clearances of lamotrigine in 7, 11, and 53 cases of women before and during pregnancy (Tran et al., 2002; Petrenaite et al., 2005; Pennell et al., 2008). By taking fertilization week as the independent variable and the sample size as weight, a cubic function describing the relative change of lamotrigine apparent clearance was fitted to these data by non-linear regression (Figure 2 and Eq. 8).

UGT1A4 activity change(%)=8.669FW−0.339FW2+0.00462FW3 (8)

#### CYP3A4

Eq. 9 describing CYP3A4 induction during pregnancy was derived based on the PBPK modeling of CYP3A4 probe substrate midazolam in pregnant women (Ke and Milad, 2019).

CYP3A4 activity (%)=1.00736+0.00564FW+0.00172FW2−0.00003FW3 (9)

#### CYP2C8

Quantitative information on CYP2C8 activity during pregnancy was not reported; therefore, CYP2C8-mediated clearance was assumed to remain unchanged in the pregnancy model.

#### FMO3

The N′-oxidation of nicotine is catalyzed solely by FMO_3_. Hukkanen et al. proposed that the ratio of urinary excretion of nicotine N′-oxide to the plasma area under the curve of nicotine could be an active indicator of FMO_3_ (excluding the effect of slightly higher urine pH during pregnancy on urinary excretion of nicotine) (Hukkanen et al., 2005). According to this study, FMO_3_ activity is increased by 58% in late pregnancy.

The setting of compound-related parameters in the pregnancy model is listed in Table 2.

**TABLE 2:** The setting of compound-related parameters in the pregnancy model. Parameters were adjusted based on their baseline values presuming 6-, 20-, and 34-weeks fertilization as representative of first, second, and third trimesters, respectively.

Parameters	1st trimester	2nd trimester	3rd trimester
f_u_	0.075	0.085	0.091
CL_int,CYP1A2_ (L/h)	22.40	14.13	9.87
CL_int,CYP3A4_ (L/h)	0.89	1.28	1.64
CL_int,CYP2C8_ (L/h)	2.14	2.14	2.14
CL_int,FMO3_ (L/h)	4.05	4.05	7.11
CL_int,UGT1A4_ (L/h)	28.24	35.04	36.91

### Pregnant Simulation

We created three virtual pregnant groups based on fertilization week ranges, including first trimester (1–11 weeks), second trimester (12–26 weeks), and third trimester (27–38 weeks), with a non-pregnant population (20–40 years old) as the reference population. Each virtual population contained 1,000 individuals. The model was applied to predict the steady-state pharmacokinetics of olanzapine in non-pregnant and pregnant women under 10 mg daily dose, which is a recommended starting and commonly used dose.

## Results

### Olanzapine PBPK Model Development

**FIGURE 3:**
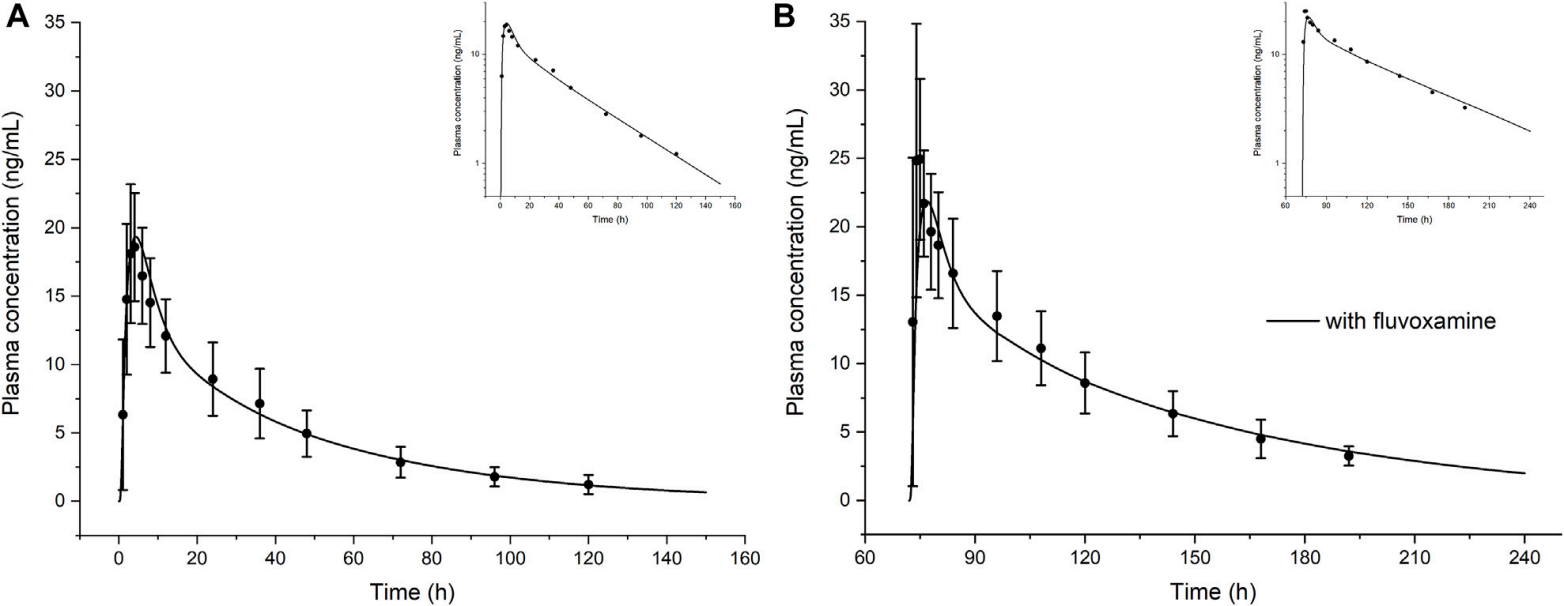
The model-simulated concentration-time curve of olanzapine after a single oral dose of 10 mg in healthy volunteers in the absence **(A)** or presence **(B)** of co-administered CYP1A2 inhibitor fluvoxamine. Curves are from the model prediction and observed mean plasma concentrations with standard deviations are shown as circles with error bars. The top right corners show the log scale figures. Figure B calculated the actual sampling time starting from day 4 that was different from the original literature.

**FIGURE 4:**
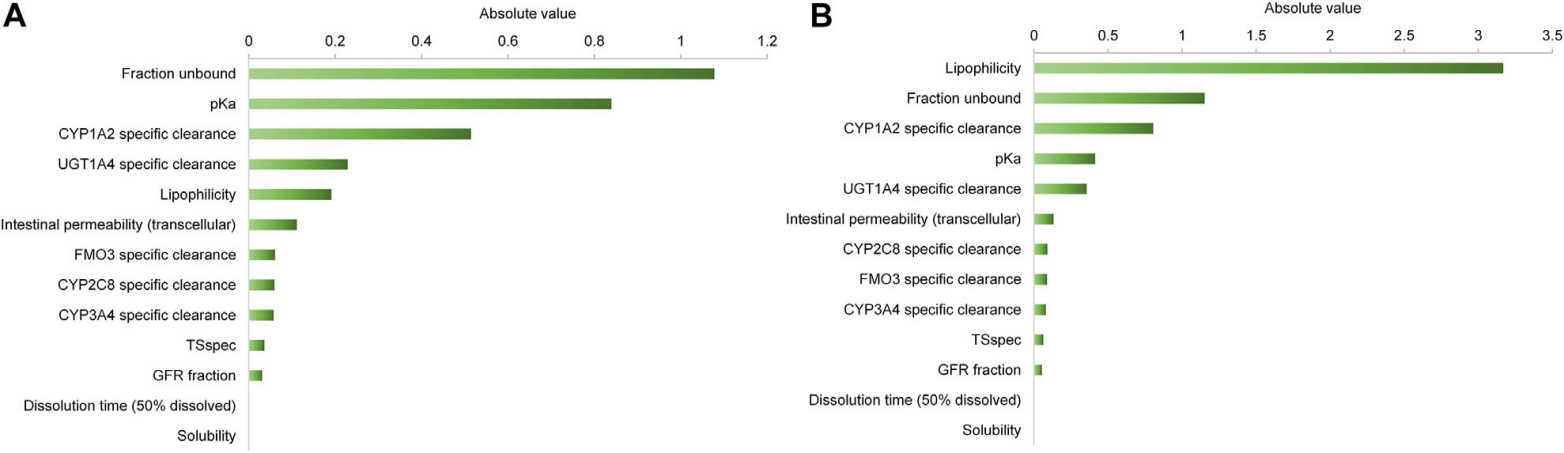
Sensitivity analysis of the olanzapine model. Sensitivity of the final model to single parameters was measured as relative change of AUC_0~∞_ after a single oral dose **(A)** or AUC from time of the last dose to infinity after multiple administrations **(B)**. A sensitivity value of +1.0 denotes that a 10% increase of the examined parameter causes a 10% increase of the simulated AUC.

As shown in Figure 3 and Table 3, the olanzapine model adequately simulates mean pharmacokinetic profiles of a 10 mg single oral dose with and without co-administration with fluvoxamine. The prediction errors for C_max_ and AUC are less than 4.5%. When co-administered with fluvoxamine, the predicted C_max_ ratio (C_max_R) and AUC ratio (AUCR) are 1.13 and 1.88, respectively, compared to the reported values of 1.34 and 1.76. The contributions of each clearance pathway have been consistent with reported values. Sensitivity analysis (Figure 4) indicates that the fraction unbound is the most sensitive parameter (−1.24) for systemic exposure to olanzapine after a single oral dose, followed by pKa (−1.02), specific clearances of CYP1A2 (−0.51) and UGT1A4 (−0.23), and lipophilicity (−0.19) among all included model parameters. As to multiple dosing, logP is the most sensitive parameter (3.17), followed by fraction unbound, CYP1A2 specific clearance, and pKa.

**TABLE 3:** Simulated and observed pharmacokinetic parameters of olanzapine from model development.

—	Single dose			Co-administered with fluvoxamine	
	C_max_ (ng/ml)	AUC_0-∞_ (ng*h/mL)	CL/F (L/h)	C_max_R	AUCR
Simulated	19.4	701.0	14.3	1.13	1.88
Reported	18.6	728.5	14.6	1.34	1.76

C_max_, peak concentration; AUC_0-∞_, area under the concentration-time curve from time zero to infinity; CL/F, apparent clearance; C_max_R, c_max_ ratio; AUCR, AUC, ratio.

### Olanzapine Model Verification

**FIGURE 5:**
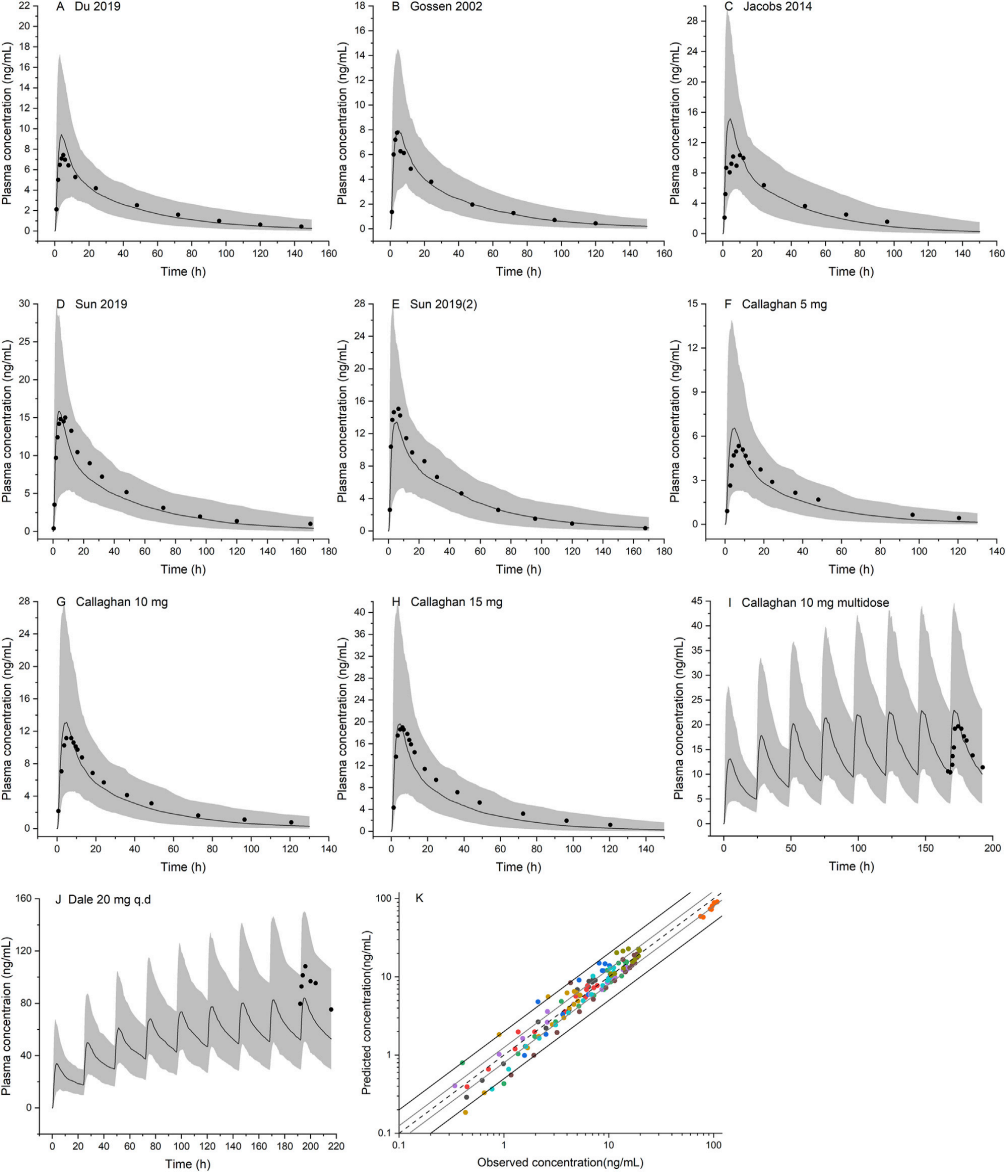
Population PBPK simulations for olanzapine in the non-pregnant population **(A–J)** Predicted median plasma concentrations are shown as dark lines, and shaded areas indicate 5th to 95th prediction range. Black dots are observed mean plasma concentrations extracted from clinical studies **(K)** Goodness of fit plot for model prediction of olanzapine plasma concentrations. Different colors represent observed-to-predicted concentration data from different simulations in figure **A–J**. The observed data are from published studies with references provided in the Supplementary Material.

Ten population PBPK simulations for the validation data set were conducted and are shown in Figure 5. More than 95% of the predicted drug concentrations are within a twofold error range of the measured values, and about 62% are within 1.25-fold error range according to the goodness-of-fit plot (Figure 5K). The mean absolute prediction errors of the plasma concentrations for all simulated studies are less than 42% (Supplementary Table S2). The fold errors for predicted/observed C_max_ and AUC are within the range of 0.75–1.30, and GMFE of C_max_ and AUC is 1.14 and 1.09, respectively (Table 4). In a pediatric simulation (Dale 2000), the model predicts a slightly lower plasma exposure in children aged 10–18.

**TABLE 4:** Predicted and observed pharmacokinetic parameters of olanzapine and geometric mean fold errors from model validation.

Study	Methods	C_max_/C_max-ss_ (ng/ml)	AUC/AUC_τ-ss_ (ng∙h/mL)	t_max_ (h)
Du et al. (2020)	Predicted	8.1	298.3	4.4
	Observed	7.8	341.4	5.0
	FE	1.04	0.87	0.88
Gossen (2002)	Predicted	7.2	264.1	4.9
	Observed	7.6	272.0	3.0
	FE	0.95	0.97	1.63
Callaghan 1999	Predicted	7.0	217.8	4.0
	Observed	5.4	236.7	7.0
	FE	1.30	0.92	0.57
Jacobs (2014)	Predicted	13.4	426.2	4.3
	Observed	13.2	436.9	6.0
	FE	1.02	0.98	0.72
Sun (2019a)	Predicted	17.2	710.3	3.9
	Observed	17.5	711.5	7.0
	FE	0.98	1.00	0.56
Sun (2019b)	Predicted	14.5	651.7	4.2
	Observed	16.7	629.2	5.0
	FE	0.87	1.04	0.84
Callaghan 1999	Predicted	14.0	436.5	4.0
	Observed	11.2	460.3	4.8
	FE	1.25	0.95	0.83
Callaghan 1999	Predicted	21.0	653.1	4.0
	Observed	19.0	755.1	6.2
	FE	1.10	0.86	0.65
Callaghan 1999	Predicted	24.8	423.1	3.7
	Observed	19.7	388.6	6.2
	FE	1.26	1.09	0.60
Dale 2000	Predicted	92.2	1731	3.0
	Observed	115.6	2,220	4.0
	FE	0.80	0.78	0.75
	GMFE	1.14	1.09	1.44

FE, fold error; GMFE, geometric mean fold error; C_max-ss_, steady-state peak concentration; AUC_τ-ss_, steady-state AUC, of a dosing interval; t_max_ time to reach peak concentration.

### Pharmacokinetic Prediction in the Pregnant Population

**FIGURE 6:**
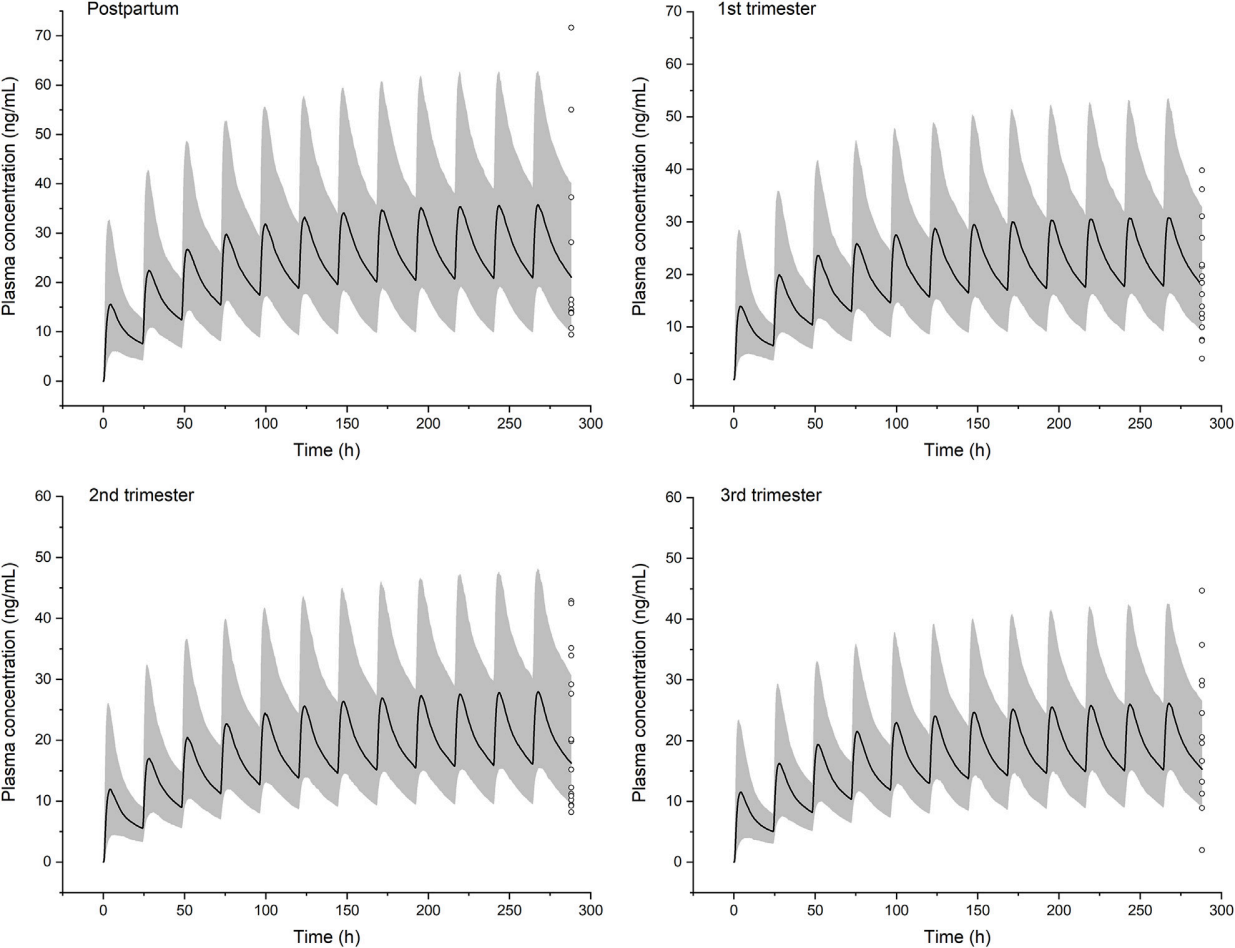
Simulated pharmacokinetic profiles of olanzapine in non-pregnant women and women in the three stages of pregnancy receiving 10 mg daily dose. Median plasma concentrations are shown as dark lines, and shaded areas indicate 5th to 95th prediction range. Circles are individual olanzapine concentration data in pregnant women with schizophrenia collected from therapeutic drug monitoring (TDM) (Westin et al., 2018). It was recommended that TDM samples are collected as trough levels at steady state.

Simulations of steady-state pharmacokinetics were performed during the first (6 weeks), second (20 weeks), and third (34 weeks) trimesters of pregnancy, in comparison with that of baseline. Fraction unbound shows a moderate increase across the first (7.1%), second (21.4%), and third (30.0%) trimester. CYP1A2 activity decreases, while UGT1A4, CYP3A4, and FMO3 are upregulated; as a result, the intrinsic clearance alters less than 20% throughout pregnancy. Overall, PBPK modeling predicts a limited impact of gestation on plasma concentrations of olanzapine (Figure 6). The fluctuation of mean plasma concentration under 10 mg daily dose is basically stable but an effective treatment concentration (20 ng/ml) cannot be guaranteed to achieve at any time in a dosing interval of late pregnancy. The steady-state C_max_, AUC_τ-ss_, and half-life of olanzapine show slight changes (not more than 28%) throughout pregnancy (Table 5). The apparent total clearance (CL/F) are increased by up to 37.1% until the late pregnancy. Based on the model prediction, the average steady-state trough concentrations of olanzapine in the first, second, and third trimester of pregnancy are decreased by 12.4, 22.6, and 28.3%, respectively. The magnitude of predicted decrease is slightly higher than the observed one calculated from TDM data. (Table 6).

**TABLE 5:** Model-predicted steady state pharmacokinetic parameters of olanzapine during pregnancy under 10 mg daily dose. Data shown as geometric means.

—	C_max-ss_ (ng/ml)	AUC_τ-ss_ (ng·h/mL)	t_1/2_ (h)	CL_ss_/F
Postpartum	35.1	653.5	32.7	15.3
1st trimester	30.5 (−13.1%)	562.9 (−11.5%)	32.5 (+0.31%)	17.8 (+16.1%)
2nd trimester	27.5 (−21.6%)	511.2 (−21.8%)	33.1 (+1.22%)	19.6 (+27.8%)
3rd trimester	25.8 (−26.5%)	476.5 (−27.1%)	33.6 (+2.75%)	21.0 (+37.1%)

C_max-ss_, steady-state peak concentration; AUC_τ-ss_, steady-state AUC of a dosing interval; t_1/2_, half life; CL_ss_/F, steady-state apparent clearance. Within brackets are relative changes during pregnancy compared to the baseline.

**TABLE 6:** The mean steady state trough concentrations of olanzapine predicted by the PBPK model and reported TDM data-based regression curve (Westin et al., 2018).

Method	BaselineConc	6 weeks gestation		20 weeks gestation		34 weeks gestation	
		Conc	Change	Conc	Change	Conc	Change
PBPK model	22.6	19.8	−12.4%	17.5	−22.6%	16.2	−28.3%
Regression curve	21.3	20.9	−1.9%	20.1	−5.6%	19.3	−9.4%

## Discussion

This study developed a PBPK model of olanzapine using a ‘middle-out’ strategy and gave pharmacokinetic predictions for the pregnant population. To our knowledge, this is the first pregnant PBPK modeling study for olanzapine.

Previous olanzapine PBPK modeling studies for non-pregnant adults described olanzapine clearance based on *in vitro* data (Polasek et al., 2018; Sun et al., 2020), while this study applied a different strategy. Because direct *in vitro* to *in vivo* extrapolation of the contribution of an enzyme to the total drug metabolism is often associated with great uncertainty (Zhang et al., 2020), we calculated this important parameter for major enzymes using *in vivo* data (mass balance and clinical DDI study). CYP1A2 has long been regarded as the primary enzyme for olanzapine metabolism, and its contribution was first determined to be 50%. Unlike typical highly polymorphic enzymes such as CYP2C9 and CYP2D6, genetic polymorphisms of CYP1A2 contribute little to the interindividual pharmacokinetic variability of olanzapine (Na Takuathung et al., 2019). Therefore, CYP1A2 polymorphisms were not considered in this study. The major circulating metabolite of olanzapine is the 10-N-glucuronide, whose formation can be attributed to UGT1A4 and UGT2B10 with the former having a much higher catalytic activity than UGT2B10 (Soderberg and Dahl, 2013). Besides, sensitivity analysis shows that fraction unbound, pKa, logP, and CYP1A2 and UGT1A4-mediated clearance are the most sensitive parameters, while logP is much more sensitive following multiple administrations. This impact leads to a visibly different time to reach the plateau under multiple doses, probably because of late back-distribution from compartments where olanzapine accumulates, that significantly affect AUC of the last dose (data not shown). As a result, a higher sensitivity to logP was observed after multiple administrations. Because the Charge-dependent Schmitt method for calculating cellular permeabilities considers the effect of electric charge, pKa becomes a relatively important parameter.

We speculated that the reduction in CYP1A2 activity during pregnancy is counteracted by the induction of other enzymes, especially UGT1A4. To this date, different studies have reported controversial results on hepatic blood flow during pregnancy. Therefore, the gestation-related physiology engine for creating virtual pregnant populations assumed unchanged absolute liver blood flow (Dallmann et al., 2017a). Alterations in CL/F should be mainly attributed to changes in (unbound fraction) x (intrinsic clearance) especially when olanzapine has a relatively low extraction ratio (<0.3). Fraction unbound, CYP1A2 and UGT1A4-mediated clearances are the most significant ones among all tested parameters modified during pregnancy (Figure 4). CYP1A2 activity decreases by up to 60% to late pregnancy. Meanwhile, UGT1A4 activity increases by 85% on average, whereas there is a slight alteration in fraction unbound, as calculated according to Eq. 5. As a result, the mean plasma concentrations of olanzapine are generally stable. On the other hand, the reliability of model predictions is potentially affected by several factors that cannot be accurately clarified at the current stage. First, the sensitivity analysis indicated that attention should be paid to the calculated fraction unbound. Although the calculation method (Eq. 5) has been evaluated for other drugs, the results stress the importance of a correct value for fraction unbound during pregnancy. For an extensively metabolized drug like olanzapine, increase in fraction unbound contributes to a higher hepatic clearance with a great possibility. Therefore, it should be beneficial to measure a precise fraction unbound of olanzapine in future clinical studies to confirm or refine the value used in the PBPK model. Second, it is currently undetermined whether gestation changes drug absorption from the gastrointestinal tract. In this model, settings for drug absorption were not specifically modified compared to non-pregnant women. Drug absorption is indeed a challenge requiring further investigation. There might be several gestation-related factors affecting drug absorption, for instance, prolongation in gastrointestinal transit time, and enlargement in intestinal villi surface area (Dallmann et al., 2018b; Koren and Pariente, 2018). However, we haven’t developed mathematical explanations for these factors due to inadequate quantitative human data. Since clinical data for C_max_/t_max_ are lacking, the simulated absorption cannot be evaluated. This stresses again the need for further clinical data during pregnancy, ideally full pharmacokinetic profiles instead of trough concentrations. Third, uncertainty in clearance contribution of some minor enzymes during model development, though we estimated it had a minimal impact. Besides, there are conflicting reports on the exact magnitude of CYP3A4 induction in pregnant women. Some studies suggest that a 2-fold induction in the third trimester is plausible, whereas others suggest a lower activity increase (such as 27%) (Nylen et al., 2011). A previous modeling study used a weighted mean of 60% induction (Dallmann et al., 2018a). Assuming a 60% activity increase in the third trimester, the model predicts a C_max_ of 26.3 ng/ml and AUC_τ-ss_ of 488.1 ngh/mL, which show negligible differences from the current data. Therefore, among all relevant enzymes, CYP1A2 and UGT1A4 activity during pregnancy are critical determinants of olanzapine clearance. More quantitative data reflecting activity changes of metabolic enzymes are needed to enhance the predictive performance of pregnant PBPK modeling.

According to the TDM data and PBPK predictions, dose adjustments appear to be not urgently needed for pregnant women. Neither has a report that pregnant women show a higher treatment failure rate under the same doses. But we should notice that TDM data have indicated a considerable interindividual variability in trough concentrations (Westin et al., 2018). Therefore, TDM has its unique strength in the individualized dosing that could not be replaced. Effective treatment before gestation is essential to olanzapine usage during pregnancy with an unchanged dosage regimen.

A limitation of this study is that fetal exposure to olanzapine has not been addressed. In order to estimate the placental transfer of drugs, data from *in vitro* cell models and *ex vivo* placental perfusion are preferred. Additionally, umbilical cord blood concentration data during delivery would be needed to validate the model predictions (Dallmann et al., 2019). Unfortunately, these pieces of information on olanzapine are lacking. Recent studies have made beneficial attempts to explore the maternal-fetal drug transfer and exposure ratio with the abovementioned approaches using acetaminophen as a model drug (Mian et al., 2020; Mian et al., 2021). These studies provide helpful references in the analysis of fetal pharmacokinetics of olanzapine in the future. We urgently need more long-term studies with large samples to clarify the efficacy and adverse impacts on fetuses and determine management strategies for antipsychotics.

## Conclusion

In summary, this study developed a PBPK model of olanzapine to evaluate the maternal exposure of this commonly prescribed antipsychotic in the pregnant population. The predictive performance was validated with various clinical pharmacokinetic studies. According to the presented PBPK simulations, the steady-state pharmacokinetics of olanzapine is slightly, and probably not clinically significantly altered during pregnancy. Combined with the TDM data, the model suggests that dose adjustment cannot be formulated for pregnant women, at least at the tested stages of pregnancy, if effective treatment was achieved before the onset of pregnancy, while fetal safety certainly needs continuous surveillance.

## Data Availability

The raw data supporting the conclusions of this article will be made available by the authors, without undue reservation.
