# Supplementary material for: Physiologically Based Pharmacokinetic Modeling in Pregnant Women Suggests Minor Decrease in Maternal Exposure to Olanzapine
Source: Front Pharmacol. 2022 Jan 19;12:793346. doi: 10.3389/fphar.2021.793346 (PMC8807508; doi:10.3389/fphar.2021.793346)
Supplement: Supplementary file 1 [file DataSheet1.docx]

Supplementary Material

# Supplementary Data

**Supplemental information on model development and verification**

This study created respective virtual individuals or populations that refer to subject demographics in each clinical study in PK-Sim®. The anatomical and physiological information of PK-Sim® default populations is from International Commission on Radiological Protection (ICRP) data without additional modification. The expression of metabolic enzymes in other tissues and organs is deduced by multiplying relative expression factor (EXPR) with liver concentration as a reference value (Equation S1).

$$C_{E,organ}=C_{E,liver}\times{EXPR}_{organ} (S1)$$

The tissue relative expressions of CYP and FMO3 refer to expression profiles (RT-qPCR) in PK-Sim® default database released with the OSP Suite (Nishimura and Naito, 2006). UGT1A4 relative expression was set 1.0 in the liver, 0.39 in the kidney, 0 in the intestine and other tissues (Harbourt et al., 2012). The default reference concentrations for enzymes were adopted. The release feature of the olanzapine tablet was simulated with a modified Weibull function (Equation S2) (50% dissolution time: 10 min).

$$m=1-exp\left( \frac{-t^{0.92}}{0.167} \right) (S2)$$

We determined the proportion of CYP1A2 clearance to the total metabolism based on a clinical drug interaction study (the test set). The study reported the effect of multiple administration of fluvoxamine, a potent CYP1A2 inhibitor, on the pharmacokinetics of olanzapine. Fluvoxamine was administered 100 mg daily for 9 consecutive days, and olanzapine was given on day 4 with a dose of 10 mg. According to the Rowland-Matin equation mentioned in the main text, f_m,CYP1A2_ was calculated as followed (Equation 1).

$$\frac{{AUC}_{i}}{AUC}=\frac{F_{g, i}}{F_{g}}\times\frac{1}{\frac{\sum f_{m}\times f_{m, CYP1A2}}{1+\frac{I_{u}}{K_{i}}}+(1-\sum f_{m}\times f_{m, CYP1A2})}$$

AUC_i_ and AUC are olanzapine AUC in the presence or absence of the inhibitor, and the reported ratio is 1.76. F_g,i_ and Fg are the proportion of effective intestinal absorption in the presence or absence of the inhibitor, respectively. CYP1A2 is predominantly expressed in the liver, and olanzapine is well absorbed through passive transport, so F_g,i_/Fg ≈ 1. Σf_m_ is the contribution of metabolism to the total clearance (0.93) (Kassahun et al., 1997). K_i_ is the inhibitory constant of fluvoxamine on CYP1A2 (10 nmol/L) (Britz et al., 2019). I_u_ is the concentration of free inhibitor in vivo calculated as the maximum free concentration of inhibitor in the portal vein during absorption, which is estimated to be 111 nmol/L by the validated PBPK model of fluvoxamine (Britz et al., 2019). A final value of 0.50 was determined for f_m,CYP1A2_.

Mathematical theory defining drug interaction processes is specified in the user tutorial of OSP Suite (https://docs.open-systems-pharmacology.org/). DDI AUC ratio (AUCR) and C_max_ ratio (C_max_ R) were calculated by Equations S3 and S4.

$$AUCR=\frac{{AUC}_{olanzapine during coadministration}}{{AUC}_{olanzapine alone}} (S3)$$

$$C_{max}R=\frac{{C_{max}}_{olanzapine during coadministration}}{{C_{max}}_{olanzapine alone}} (S4)$$

In model validation, each created virtual population contains 200 individuals. The virtual populations applied in population simulations were created using the PK-Sim Population module. Demographic characteristics of virtual populations, such as the distribution of age, sex, and weight, are comparable to the observed data. The subject demographics and dosing information are listed in Table S1. Half of the subjects from Callahgan et al. are smokers (Callaghan et al., 1999). CYP1A2 enzyme expression in heavy smokers was induced by three times compared to nonsmokers (Sun et al., 2020). For modeling smokers, rather than modifying CYP1A2 concentration, we adjusted CYP1A2-mediated clearances as it is mathematically exchangeable.

Apart from GMFE mentioned in the main text, the mean absolute prediction error (MAPE) was calculated to evaluate the predictive performance according to Equation S3.

$$PE=\frac{pred conc-obs conc}{obs conc}\times100\% MAPE(\%)=\frac{1}{n}\sum_{i=1}^{n} \left| {PE}_{i} \right|\text{ (}\text{S5}\text{)}$$

where *pred conc* is the predicted concentration, and *obs conc* is the corresponding observed concentration. MAPEs for all validation sets are less than 27%, indicating a satisfactory predictive performance (Table S2).

# Supplementary Figures and Tables

## Supplementary Figures

**Supplementary Figure 1.** In vivo biotransformation of olanzapine. UGT1A4 catalyzes 10-N-glucuronidation and 4’-N-glucuronidation whose metabolites account for 23% of an administered oral dose. The precise proportions of other metabolites produced by oxidases have not been determined.

## Supplementary Tables

**Supplementary Table 1.** Summary of pharmacokinetic reports of olanzapine used in PBPK modeling.

| Study | Ethics | Age, mean ± SD (range) | Number of subjects | Health/disease status | Proportion of female | Dosing regimen | References |
| --- | --- | --- | --- | --- | --- | --- | --- |
| **Test set** | | | | | | | |
| Wang 2004 | Asian | 26.7 ± 4.3 | 21 | healthy nonsmoker | 0 | 10 mg p.o.  single dose | (Wang et al., 2004) |
| Wang 2004 | Asian | 26.7 ± 4.3 | 21 | healthy nonsmoker | 0 | 10 mg p.o. on day 4 with fluvoxamine 100 mg daily for 9 days | (Wang et al., 2004) |
| **Validation set** | | | | | | | |
| Callaghan 1999 | Caucasian | 30 (19 ~ 79) | NA | healthy  50% smoker | 0 | 5 mg single dose | (Callaghan et al., 1999) |
| Callaghan 1999 | Caucasian | 30 (19 ~ 79) | NA | healthy  50% smoker | 0 | 10 mg single dose | (Callaghan et al., 1999) |
| Callaghan 1999 | Caucasian | 30 (19 ~ 79) | NA | healthy  50% smoker | 0 | 15 mg single dose | (Callaghan et al., 1999) |
| Callaghan 1999 | Caucasian | 30 (19 ~ 79) | NA | healthy  50% smoker | 0 | 10 mg multidose steady-state | (Callaghan et al., 1999) |
| Gossen 2002 | Caucasian/Hispanic | 32 ± 5 | 15 | healthy nonsmoker | 27% | 5 mg single dose | (Gossen et al., 2002) |
| Jacobs 2014 | Caucasian | 42 (25.8 ~ 49.8) | 24 | healthy nonsmoker | 54% | 10 mg single dose | (Jacobs et al., 2014) |
| Du 2019 | Asian | 30 ± 6 | 30 | healthy nonsmoker | 13% | 5 mg single dose | (Du et al., 2020) |
| Sun 2019 (1) | White American | 31 (20 ~ 40) | 24 | healthy nonsmoker | 54% | 10 mg single dose | (Sun et al., 2019b) |
| Sun 2019 (2) | Black American | 26.7 ± 4.9 | 48 | healthy nonsmoker | 42% | 10 mg single dose | (Sun et al., 2019a) |
| Dale 2000 | White American | 10 ~ 18 | 8 | schizophrenic children and adolescents | 50% | 20 mg multidose steady-state | (Grothe et al., 2000) |

**Supplementary Table 2.** Mean absolute prediction errors for plasma olanzapine concentrations.

| Study | MAPE (%) |
| --- | --- |
| Du 2019 (5 mg) | 23.0 |
| Gossen 2002 (5 mg) | 11.5 |
| Callaghan 1999 (5 mg) | 36.3 |
| Jacobs 2014 (10 mg) | 41.9 |
| Sun 2019 (1) (10 mg) | 24.8 |
| Sun 2019 (2) (10 mg) | 13.4 |
| Callaghan 1999 (10 mg) | 19.9 |
| Callaghan 1999 (15 mg) | 26.6 |
| Callaghan 1999 (10 mg multidose) | 19.8 |
| Dale 2000 (20 mg multi-dose) | 19.6 |

**References**

Britz, H., Hanke, N., Volz, A.K., Spigset, O., Schwab, M., Eissing, T., et al. (2019). Physiologically-Based Pharmacokinetic Models for CYP1A2 Drug-Drug Interaction Prediction: A Modeling Network of Fluvoxamine, Theophylline, Caffeine, Rifampicin, and Midazolam. *CPT Pharmacometrics Syst Pharmacol* 8(5)**,** 296-307.

Callaghan, J.T., Bergstrom, R.F., Ptak, L.R., and Beasley, C.M. (1999). Olanzapine. Pharmacokinetic and pharmacodynamic profile. *Clin Pharmacokinet* 37(3)**,** 177-193. doi: 10.2165/00003088-199937030-00001.

Du, P., Li, P., Liu, H., Zhao, R., Zhao, Z., Yu, W., et al. (2020). Open-Label, Randomized, Single-Dose, 2-Period, 2-Sequence Crossover, Comparative Pharmacokinetic Study to Evaluate Bioequivalence of 2 Oral Formulations of Olanzapine Under Fasting and Fed Conditions. *Clin Pharmacol Drug Dev* 9(5)**,** 621-628. doi: 10.1002/cpdd.743.

Gossen, D., de Suray, J.M., Vandenhende, F., Onkelinx, C., and Gangji, D. (2002). Influence of fluoxetine on olanzapine pharmacokinetics. *AAPS PharmSci* 4(2)**,** E11. doi: 10.1208/ps040211.

Grothe, D.R., Calis, K.A., Jacobsen, L., Kumra, S., DeVane, C.L., Rapoport, J.L., et al. (2000). Olanzapine pharmacokinetics in pediatric and adolescent inpatients with childhood-onset schizophrenia. *J Clin Psychopharmacol* 20(2)**,** 220-225. doi: 10.1097/00004714-200004000-00015.

Harbourt, D.E., Fallon, J.K., Ito, S., Baba, T., Ritter, J.K., Glish, G.L., et al. (2012). Quantification of human uridine-diphosphate glucuronosyl transferase 1A isoforms in liver, intestine, and kidney using nanobore liquid chromatography-tandem mass spectrometry. *Anal Chem* 84(1)**,** 98-105. doi: 10.1021/ac201704a.

Jacobs, B.S., Colbers, A.P., Velthoven-Graafland, K., Schouwenberg, B.J., and Burger, D.M. (2014). Effect of fosamprenavir/ritonavir on the pharmacokinetics of single-dose olanzapine in healthy volunteers. *Int J Antimicrob Agents* 44(2)**,** 173-177. doi: 10.1016/j.ijantimicag.2014.03.014.

Kassahun, K., Mattiuz, E., Nyhart, E., Jr., Obermeyer, B., Gillespie, T., Murphy, A., et al. (1997). Disposition and biotransformation of the antipsychotic agent olanzapine in humans. *Drug Metab Dispos* 25(1)**,** 81-93.

Nishimura, M., and Naito, S. (2006). Tissue-specific mRNA expression profiles of human phase I metabolizing enzymes except for cytochrome P450 and phase II metabolizing enzymes. *Drug Metab Pharmacokinet* 21(5)**,** 357-374. doi: 10.2133/dmpk.21.357.

Sun, L., McDonnell, D., Liu, J., and von Moltke, L. (2019a). Bioequivalence of Olanzapine Given in Combination With Samidorphan as a Bilayer Tablet (ALKS 3831) Compared With Olanzapine-Alone Tablets: Results From a Randomized, Crossover Relative Bioavailability Study. *Clin Pharmacol Drug Dev* 8(4)**,** 459-466. doi: 10.1002/cpdd.601.

Sun, L., McDonnell, D., Yu, M., Kumar, V., and von Moltke, L. (2019b). A Phase I Open-Label Study to Evaluate the Effects of Rifampin on the Pharmacokinetics of Olanzapine and Samidorphan Administered in Combination in Healthy Human Subjects. *Clin Drug Investig* 39(5)**,** 477-484. doi: 10.1007/s40261-019-00775-8.

Sun, L., von Moltke, L., and Yeo, K.R. (2020). Physiologically-Based Pharmacokinetic Modeling for Predicting Drug Interactions of a Combination of Olanzapine and Samidorphan. *Cpt-Pharmacometrics & Systems Pharmacology* 9(2)**,** 106-114. doi: 10.1002/psp4.12488.

Wang, C.Y., Zhang, Z.J., Li, W.B., Zhai, Y.M., Cai, Z.J., Weng, Y.Z., et al. (2004). The differential effects of steady-state fluvoxamine on the pharmacokinetics of olanzapine and clozapine in healthy volunteers. *J Clin Pharmacol* 44(7)**,** 785-792. doi: 10.1177/0091270004266621.
